# Supplementary material for: Two receptor tyrosine phosphatases dictate the depth of axonal stabilizing layer in the visual system
Source: eLife. 2017 Nov 8;6:e31812. doi: 10.7554/eLife.31812 (PMC5683756; doi:10.7554/eLife.31812)
Supplement: Supplementary file 1. [file elife-31812-supp1.docx]

**Supplementary Table 1 　List of all full genotypes used**

| Figure | Genotype (Genotypes unrelated to the context are in brackets.) |
| --- | --- |
| 1A, 1E | w; <LAR<, Rh4-mCD8GFP/+; <Ptp69D<, Rh4-mCD8GFP/+ |
| 1B | yw, eyFLP2 (c-lacZ)/w; LAR[2127], Rh4-mCD8GFP/<LAR<, Rh4-mCD8GFP |
| 1C | yw, eyFLP2, Rh4-mCD8GFP/w; Ptp69D[D1689] Rh4-mCD8GFP/<Ptp69D< |
| 1D, 1F | yw, eyFLP2 (c-lacZ)/w; LAR[2127] /<LAR<, Rh4-mCD8GFP; Ptp69D[D1689] /<Ptp69D<, Rh4-mCD8GFP |
| 2A-2G | w; LAR[2127], 20C11-FLP, GMR-Gal4/UAS-LARRNAi, UAS-Ptp69DRNAi; Ptp69D[D1689]/UAS-FRT-stop-FRT-mCD8GFP |
| 3A-3C | Rh4-mCD8GFP; GMR-Gal4 tub-Gal80[ts] /UAS-LARRNAi, UAS-Ptp69DRNAi; |
| 3D, 3E | Rh4-mCD8GFP/+; LAR[2127],GMR-Gal4/UAS-LARRNAi, UAS-Ptp69DRNAi; Ptp69D[D1689] , Rh4-mCD8GFP/+ |
| 4A | Same as 1D |
| 4B | yw, eyFLP2 (c-lacZ)/w; LAR[2127], GMR-Gal4/<LAR<, Rh4-mCD8GFP; Ptp69D[D1689], Rh4-mCD8GFP /<Ptp69D< UAS-LARFL |
| 4C | yw, eyFLP2 (c-lacZ)/w; LAR[2127], GMR-Gal4/<LAR<, Rh4-mCD8GFP; Ptp69D[D1689], Rh4-mCD8GFP /<Ptp69D< UAS-LARΔC |
| 4D | yw, eyFLP2 (c-lacZ)/w; LAR[2127], GMR-Gal4/<LAR<, Rh4-mCD8GFP; Ptp69D[D1689], Rh4-mCD8GFP /<Ptp69D< UAS-LARD1CS |
| 4E | yw, eyFLP2 (c-lacZ)/w; LAR[2127], GMR-Gal4/<LAR<, Rh4-mCD8GFP; Ptp69D[D1689], Rh4-mCD8GFP /<Ptp69D< UAS-LAR2XCS |
| 4F | yw, eyFLP2 (c-lacZ)/w; LAR[2127], GMR-Gal4/<LAR<, Rh4-mCD8GFP; Ptp69D[D1689], Rh4-mCD8GFP /<Ptp69D< UAS-LARΔFn7-9 |
| 4G | yw, eyFLP2 (c-lacZ)/w; LAR[2127], GMR-Gal4/<LAR<, UAS-LARΔIg1-3; Ptp69D[D1689], Rh4-mCD8GFP /<Ptp69D< Rh4-mCD8GFP |
| 4H | yw, eyFLP2 (c-lacZ)/w; LAR[2127], GMR-Gal4/<LAR<, Rh4-mCD8GFP; Ptp69D[D1689], Rh4-mCD8GFP /<Ptp69D<, UAS-LARΔIg1-3ΔFn1-6 |
| 4I | yw, eyFLP2 (c-lacZ)/w; LAR[2127], GMR-Gal4/<LAR<, UAS-Ptp69DFL; Ptp69D[D1689], Rh4-mCD8GFP /<Ptp69D< |
| 4J | yw, eyFLP2 (c-lacZ)/ UAS-Ptp69DΔintra; LAR[2127], GMR-Gal4 /<LAR<, Rh4-mCD8GFP; Ptp69D[D1689] /<Ptp69D<, Rh4-mCD8GFP |
| 4K | yw, eyFLP2 (c-lacZ)/w; LAR[2127], GMR-Gal4/<LAR<; Ptp69D[D1689], Rh4-mCD8GFP /<Ptp69D< UAS-Ptp69DDA1 |
| 4L | yw, eyFLP2 (c-lacZ)/ UAS-Ptp69DDA1DA2; LAR[2127], GMR-Gal4 /<LAR<, Rh4-mCD8GFP; Ptp69D[D1689] , Rh4-mCD8GFP /<Ptp69D< |
| 4M DM+LAR* | yw, eyFLP2 (c-lacZ)/w; LAR[2127], GMR-Gal4/<LAR<, Rh4-mCD8GFP; Ptp69D[D1689], Rh4-mCD8GFP /<Ptp69D< UAS-LAR* (Only UAS-LARΔIg1-3 is on the second. The genotype for it is shown in 4G) |
| 4M PtpM+LarFL | yw, eyFLP2 (c-lacZ)/w; LAR[2127], Rh4-mCD8GFP/<LAR<, Rh4-mCD8GFP; UAS-LARFL/+ |
| 4N DM+Ptp* | Same as 4G-4J |
| 4N LARM+PtpFL | yw, eyFLP2 (c-lacZ)/w; LAR[2127], GMR-Gal4/<LAR<, UAS-Ptp69DFL; Rh4-mCD8GFP /+ |
| 5A | w; <LAR<, Rh6-mCD8GFP/+; <Ptp69D<, Rh6-mCD8GFP/+ |
| 5B | yw, eyFLP2 (c-lacZ)/w; LAR[2127]/<LAR<, Rh6-mCD8GFP; Ptp69D[D1689] /<Ptp69D<, Rh6-mCD8GFP |
| 5C | yw, eyFLP2 (c-lacZ)/w; LAR[2127]/<LAR<, Rh6-mCD8GFP; Ptp69D[D1689] , Rh6-mCD8GFP /<Ptp69D<, UAS-LARFL |
| 5D | yw, eyFLP2 (c-lacZ)/w; LAR[2127]/<LAR<, Rh6-mCD8GFP; Ptp69D[D1689] , Rh6-mCD8GFP /<Ptp69D<, UAS-LAR2XCS |
| 5E | yw, eyFLP2 (c-lacZ)/w; LAR[2127], GMR-Gal4/<LAR<, UAS-Ptp69DFL; Ptp69D[D1689], Rh6-mCD8GFP /<Ptp69D< |
| 5F | yw, eyFLP2 (c-lacZ)/ UAS-Ptp69DDA1DA2; LAR[2127], GMR-Gal4 /<LAR<, Rh6-mCD8GFP; Ptp69D[D1689] , Rh6-mCD8GFP /<Ptp69D< |
| 5G DM+LAR* | yw, eyFLP2 (c-lacZ)/w; LAR[2127]/<LAR<, Rh6-mCD8GFP; Ptp69D[D1689] , Rh6-mCD8GFP /<Ptp69D<, UAS-LAR* |
| 5G DM+PtpΔintra | yw, eyFLP2 (c-lacZ)/ UAS-Ptp69DΔintra; LAR[2127], GMR-Gal4 /<LAR<, Rh6-mCD8GFP; Ptp69D[D1689] /<Ptp69D<, Rh6-mCD8GFP |
| 5G DM+PtpDA1DA2 | Same as 5F |
| 5G DM+PtpDA1 | yw, eyFLP2 (c-lacZ)/w; LAR[2127], GMR-Gal4 /<LAR<; Ptp69D[D1689], Rh6-mCD8GFP /<Ptp69D< UAS-Ptp69DDA1 |
| 5G DM+Ptp69DFL | Same as 5E |
| 5G LARM | yw, eyFLP2 (c-lacZ)/w; LAR[2127] /<LAR<, Rh4-mCD8GFP |
| 5G PtpM | yw, eyFLP2 /w; Ptp69D[D1689] Rh4-mCD8GFP/<Ptp69D< |
| 6A | Same as 1B |
| 6B | yw, eyFLP2 (c-lacZ)/w; LAR[2127], GMR-Gal4/<LAR<, Rh4-mCD8GFP; UAS-LARΔC/+ |
| 6C | yw, eyFLP2 (c-lacZ)/ UAS-Ptp69DΔintra; LAR[2127], GMR-Gal4/<LAR<; Rh4-mCD8GFP/+ |
| 6D | Same as 1C |
| 6E | yw, eyFLP2, Rh4-mCD8GFP/w; GMR-Gal4/+ Ptp69D[D1689] Rh4-mCD8GFP/<Ptp69D< UAS-LARΔC |
| 6F | yw, eyFLP2 (c-lacZ)/UAS-Ptp69DΔintra; GMR-Gal4/+ Ptp69D[D1689] Rh4-mCD8GFP/<Ptp69D< |
| 6G LARM+LARFL | yw, eyFLP2 (c-lacZ)/w; LAR[2127], GMR-Gal4/<LAR<, Rh4-mCD8GFP; UAS-LARFL/+ |
| 6G LARM+PtpFL | yw, eyFLP2 (c-lacZ)/w; LAR[2127], GMR-Gal4/<LAR<, UAS-Ptp69DFL ; Rh4-mCD8GFP /+ |
| 6G PtpM+LARFL | yw, eyFLP2, Rh4-mCD8GFP/w; GMR-Gal4/+ Ptp69D[D1689] Rh4-mCD8GFP/<Ptp69D< UAS-LARFL |
| 6G PtpM+PtpFL | yw, eyFLP2, Rh4-mCD8GFP/w; GMR-Gal4/UAS-Ptp69DFL; Ptp69D[D1689] Rh4-mCD8GFP/<Ptp69D< |
| 6G + LARΔC | Rh4-mCD8GFP/+; GMR-Gal4/+, UAS-LARΔC/+ |
| 6G + PtpΔintra | Rh4-mCD8GFP/ UAS-Ptp69DΔintra; GMR-Gal4/+ |
| 7A R7 clone control | GMR-FLP, UAS-mCD8GFP/+; GMR-Gal4/+; (ato-τmyc) FRT80/tub-Gal80, GMR-mCD8KOmyc, FRT80 |
| 7B double mutant R7 clone | GMR-FLP, UAS-mCD8GFP/+; LAR[2127], GMR-Gal4/ UAS-LARRNAi,; Ptp69D[D1689], FRT80/tub-Gal80, GMR-mCD8KOmyc, FRT80 |
| 7D double mutant | Same as 1D |
| 7E R7 specific rescue | yw, eyFLP2 (c-lacZ)/PM181-Gal4; LAR[2127] /<LAR<, Rh4-mCD8GFP; Ptp69D[D1689] /<Ptp69D< UAS-LARFL |
| 7G double mutant | Same as 5B |
| 7H R8 specific rescue | yw, eyFLP2 (c-lacZ)/2-80Gal4; LAR[2127] /<LAR<, Rh6-mCD8GFP; Ptp69D[D1689] /<Ptp69D< UAS-LARFL |
| 8A | Rh4-mCD8GFP/Y; GMR-Gal4/UAS-LARRNAi, UAS-Ptp69DRNAi |
| 8B | Rh4-mCD8GFP/Y; GMR-Gal4/UAS-LARRNAi, UAS-Ptp69DRNAi; UAS-AblRNAi |
| 8C | Rh4-mCD8GFP/Y; GMR-Gal4/UAS-LARRNAi, UAS-Ptp69DRNAi; UAS-Abl |
| 8D | Rh4-mCD8GFP/Y; GMR-Gal4/UAS-LARRNAi, UAS-Ptp69DRNAi; UAS-EnaRNAi |
| 8E | Rh4-mCD8GFP/Y; GMR-Gal4/UAS-LARRNAi, UAS-Ptp69DRNAi; UAS-TrioRNAi |
| 8F | Rh4-mCD8GFP/Y; GMR-Gal4/UAS-LARRNAi, UAS-Ptp69DRNAi; UAS-Trio |
| 8G | Rh4-mCD8GFP/Y; GMR-Gal4/UAS-LARRNAi, UAS-Ptp69DRNAi; UAS-Ena |
| Fig.2-fig.suppl.1 | Rh4-mCD8GFP; GMR-Gal4 tub-Gal80[ts] /UAS-LARRNAi, UAS-Ptp69DRNAi; |
| Fig.3-fig.suppl.1 | Control: w  Level5-3: GMR-Gal4 tub-Gal80[ts] /UAS-LARRNAi, UAS-Ptp69DRNAi;+  Level2-1: (20C11FLP) LAR[2127],GMR-Gal4/UAS-LARRNAi, UAS-Ptp69DRNAi; Ptp69D[D1689]/ (UAS-FRT-stop-FRT-mCD8GFP) |
| Fig.4-fig.suppl.1 | Control: GMR-myrRFP  A: GMR-myrRFP/+; GMR-Gal4 UAS-LAR*(for transgenes on II) or GMR-myrRFP/+; GMR-Gal4/+; UAS-LAR*/+ (for transgenes on III)  B: GMR-myrRFP/ UAS-Ptp69D* (for Δintra and DA1DA2 on X); GMR-Gal4/+, GMR-myrRFP/+; GMR-Gal4 UAS- Ptp69DFL or GMR-myrRFP/+; GMR-Gal4/+; UAS-Ptp69DDA1/+ |
| Fig.4-fig.suppl.2 | w |
| Fig.5-fig.suppl.1A | Rh6-mCD8GFP/+; GMR-Gal4 /+; UAS-LAR/+ |
| Fig.5-fig.suppl.1B | Rh6-mCD8GFP/+; GMR-Gal4 /+; UAS-Ptp69D/+ |
| Fig.6-fig.suppl.1A | Rh4-mCD8GFP; GMR-Gal4 /+; UAS-LAR/+ |
| Fig.6-fig.suppl.1B | Rh4-mCD8GFP; GMR-Gal4 /+; UAS-Ptp69D/+ |
| Fig.7-fig.suppl.1A | PM181-Gal4/+; UAS-mCD8GFP/+ |
| Fig.7-fig.suppl.1B | 2-80-Gal4/+; UAS-mCD8GFP/+ |
| Fig.8-fig.suppl.1B | Rh4-mCD8GFP/Y; GMR-Gal4/U+; UAS-EnaRNAi |
| Fig.8-fig.suppl.1C | Rh4-mCD8GFP/Y; GMR-Gal4/+; UAS-TrioRNAi |
| Fig.8-fig.suppl.1D | Rh4-mCD8GFP/Y; GMR-Gal4+; UAS-Abl |
| Fig.8-fig.suppl.1E | Rh4-mCD8GFP/Y; GMR-Gal4/+; UAS-Ena |
| Fig.8-fig.suppl.1F | Rh4-mCD8GFP/Y; GMR-Gal4/+; UAS-Trio |
| Fig.9-fig.suppl.1A | Same as 1A |
| Fig.9-fig.suppl.1B | Rh4-mCD8GFP/+; LAR[2127],GMR-Gal4/UAS-LARRNAi, UAS-Ptp69DRNAi; Ptp69D[D1689] , Rh4-mCD8GFP/+ |
| Fig.9-fig.suppl.1C | Rh4-mCD8GFP/+; LAR[2127],GMR-Gal4/UAS-LARRNAi, UAS-Ptp69DRNAi; Ptp69D[D1689] , Rh4-mCD8GFP/UAS-TrpA1 |
